# PhyloMap: an algorithm for visualizing relationships of large sequence data sets and its application to the influenza A virus genome

**DOI:** 10.1186/1471-2105-12-248

**Published:** 2011-06-20

**Authors:** Jiajie Zhang, Amir Madany Mamlouk, Thomas Martinetz, Suhua Chang, Jing Wang, Rolf Hilgenfeld

**Affiliations:** 1 Institute of Biochemistry, Center for Structural and Cell Biology in Medicine, University of Lübeck, Ratzeburger Allee 160, 23538 Lübeck, Germany; 2 Graduate School for Computing in Medicine and Life Sciences, University of Lübeck, Ratzeburger Allee 160, 23538 Lübeck, Germany; 3 Institute for Neuro- and Bioinformatics, University of Lübeck, Ratzeburger Allee 160, 23538 Lübeck, Germany; 4 Key Laboratory of Mental Health, Institute of Psychology, Chinese Academy of Sciences, Beijing 100101, China; 5 Laboratory for Structural Biology of Infection and Inflammation, c/o DESY, Building 22a, Notkestr. 85, 22603 Hamburg, Germany; 6 Shanghai Institute of Materia Medica, Chinese Academy of Sciences, 555 Zu Chong Zhi Rd., Shanghai 201203, China

## Abstract

**Background:**

Results of phylogenetic analysis are often visualized as phylogenetic trees. Such a tree can typically only include up to a few hundred sequences. When more than a few thousand sequences are to be included, analyzing the phylogenetic relationships among them becomes a challenging task. The recent frequent outbreaks of influenza A viruses have resulted in the rapid accumulation of corresponding genome sequences. Currently, there are more than 7500 influenza A virus genomes in the database. There are no efficient ways of representing this huge data set as a whole, thus preventing a further understanding of the diversity of the influenza A virus genome.

**Results:**

Here we present a new algorithm, "PhyloMap", which combines ordination, vector quantization, and phylogenetic tree construction to give an elegant representation of a large sequence data set. The use of PhyloMap on influenza A virus genome sequences reveals the phylogenetic relationships of the internal genes that cannot be seen when only a subset of sequences are analyzed.

**Conclusions:**

The application of PhyloMap to influenza A virus genome data shows that it is a robust algorithm for analyzing large sequence data sets. It utilizes the entire data set, minimizes bias, and provides intuitive visualization. PhyloMap is implemented in JAVA, and the source code is freely available at http://www.biochem.uni-luebeck.de/public/software/phylomap.html

## Background

Phylogenetic trees are commonly used as a visualization tool [1] to help reveal the relationships among homologous sequences. When the number of sequences is limited, the relationships can be clearly observed from the tree; however, when more than a few thousand sequences are to be included, not only the accuracy of the inferred phylogenetic trees decreases, but it also becomes increasingly difficult to study the resulting trees and find patterns [2], and the computational demands of building a huge phylogenetic tree tend to be staggering. Researchers usually build a tree by sampling a small amount of data rather than constructing a complete tree using the entire dataset [3-8]. However, the sampling is generally done according to the experience of the researcher and is sometimes arbitrary. The conclusions drawn from such trees may be biased.

Higgins used Principal Coordinate Analysis (PCoA) [9] to visualize large sequence data sets, which are difficult to analyze using phylogenetic trees. He showed that PCoA can be considered complementary to phylogenetic tree analysis as it does not assume an underlying hierarchical structure in the data. A similar multidimensional scaling method was used by Smith et al [10] to analyze the antigenic and genetic evolution of influenza A virus. Wong et al. [11] used correspondence analysis to show the codon usage biases of influenza A virus. Ordination (i.e. displaying a set of data points in two or three dimensions so as to make the relationships among the points in higher dimensional space visible) has proved to be a powerful tool to visualize large datasets with high dimensionalities; nevertheless, it only preserves the main trends in the data but most of the information on detail gets lost. When the intrinsic dimensions of the data set are high, the results can sometimes be misleading.

Here, we present a new method - Phylogenetic Map (PhyloMap) - that combines PCoA, vector quantization, and phylogenetic tree construction to give an elegant visualization of a large sequence data set using all the data while still trying to capture the accurate relationships among them. Compared to traditional phylogenetic tree analysis, which is practicable only with a maximum of a few hundred sequences, PhyloMap can handle thousands of sequences at one time. PhyloMap first uses PCoA to help depict the main trends and then uses the "Neural-Gas" approach [12] to obtain multiple data centers which best represent the data set. The resulting data centers will be used to build a phylogenetic tree. Finally, we map the tree onto the PCoA result by preserving the tree topology and the distances. As the two different visualizations are superimposed, the resulting plot can greatly reduce the risk of misinterpretation.

Influenza A viruses are commonly classified by serological differences in their hemagglutinin (HA) and neuraminidase (NA) proteins. The gene sequences between different HAs or NAs are also significantly divergent and can be easily classified by serological type. However, the recent emergence of the 2009 H1N1 swine-origin human influenza A (H1N1) virus (S-OIV) [13] demonstrates that this classification has its limitations: "H1N1" is the designation for one of the two established seasonal subtypes as well as for the highly pathogenic 1918 virus that caused the "Spanish flu" pandemic [14-20], and for the currently spreading new swine-origin virus [5]. While a better classification is obviously needed [3,21], the cluster patterns of the internal genes (PB2, PB1, PA, NP, M1, M2, NS1, and NS2) of influenza A virus are less clear. We applied PhyloMap to influenza A virus internal genes, using all publicly available sequences. The results reveal patterns in those genes that cannot be seen when only a subset of sequences is analyzed, and can help us better characterize the diversity of influenza A virus genomes by considering not only the serological type differences but also the internal genes.

## Methods

### The PhyloMap algorithm

**Figure 1:**
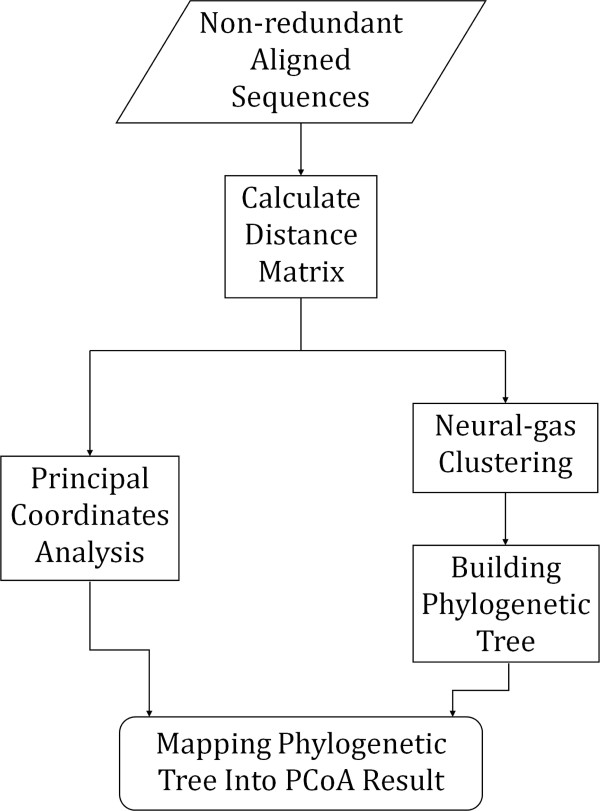
**Flow chart of the PhyloMap algorithm**.

The input to PhyloMap is a set of aligned sequences, either amino acids or nucleotides. The algorithm involves five steps as shown in Figure 1. First, a distance matrix is calculated using the input alignment. This distance matrix will serve as the input to PCoA and Neural-Gas to get the principal coordinates of each sequence and *k *sequences as cluster centers, where *k *is defined by the user. Subsequently, the *k *sequences selected by the clustering algorithm will be used to build a phylogenetic tree. Finally, we adopted a multidimensional scaling technique similar to "Sammon's mapping" [22] to map the phylogenetic tree onto the first two axes of the principal coordinates. The results can then be plotted for inspection.

#### 1. Distance Matrix

The idea of ordination is to map the input sequences onto a low-dimensional space so that the distances and relationships of the sequence set are preserved as much as possible. In order to do that, one has to calculate a distance matrix *D *which contains the distances between each pair of sequences. The distance matrix is calculated by the "Phylip" package [23] using a continuous-time Markov process. Higgins [9] suggested several ways of calculating distances that will be guaranteed to be Euclidean such as the simple P-distance and using the Smith & Smith matrix [24]. However, none of these measurements can correct multiple substitutions, and they do not follow any evolutionary model. The distances inferred by the continuous-time Markov process [25] are not Euclidean but are close to P-distance when the sequence divergence is small. As the purpose of PCoA is to find the main trends rather than accurately reconstruct the distances between sequences in the lower dimensional space, the effect of non-Euclidean distances can be neglected. For the influenza A internal protein sequences analyzed here, the Jones-Taylor-Thornton [26] model is used to infer the distances.

#### 2. Principal coordinate analysis

PCoA was first described by Gower [27]. Starting by converting the *n *× *n *distance matrix *D*, which has elements *d*_*ij*_, to the similarity matrix *E *with elements(1)

*E *is then centralized so that we have matrix *F *with elements(2)

where  is the mean of row *i*,  is the mean of column *j*, and  is the grand mean of the matrix *E*.

The eigenvectors and the eigenvalues of the matrix *F *are calculated. Each eigenvector is normalized so that its sum of squares equals the corresponding eigenvalue. The eigenvectors are ranked according to the eigenvalue in a decreasing order. The first two eigenvectors are used as the two-dimensional coordinates of each sequence. The information (variation) preserved by the first two eigenvectors is the ratio of the sum of the first two eigenvalues to the sum of all eigenvalues.

#### 3. Vector quantization (Clustering)

The clustering algorithm we choose here is the "Neural-Gas" [12]. The Neural-Gas proceeds similar to k-means but has the nice feature of providing results which hardly depend on the initialization. Therefore, performing only one run is sufficient and the algorithm yields stable results when run multiple times. The output of the clustering algorithm is a set of *k *cluster centers, where *k *is defined by the user. The Neural-Gas provides cluster centers each of which minimize the mean distance to the sequences it represents. However, we are not really searching for clusters. What we want is a set of sequences that best represent the data set. Therefore, finally, we substitute each cluster center by its closest sequence. The Neural-Gas will also guarantee that the centers are evenly distributed across the entire data set. In this application of Neural-Gas, we consider the algorithm as a sampling rather than as a clustering method. When using the resulting center sequences to build a phylogenetic tree, the tree will explore the variation of the data set without bias. For details of the algorithm, please refer to Martinetz *et al. *[12]. The number of sampling sequences might influence the accuracy of the inferred phylogenetic tree (see Discussion). For visualization purposes, it should not be too low, or else the sampling sequences would not be sufficient to represent the variation of the data. If chosen too high, the result of PhyloMap might be difficult to inspect visually. In practice, we found a sampling tree with no more than 50 sequences can be shown clearly in PhyloMap.

#### 4. Phylogenetic tree construction

Subsequently, we use the sequences selected by the Neural-Gas to build a phylogenetic tree. The Neighbor-joining (NJ) tree is used in PhyloMap with the same distance measurement used for calculating the distance matrix for PCoA. Other non-distance-based tree building methods can also be used (see the discussion below). The NJ tree is unrooted since we just want to find the major lineages of the sequences rather than to portray the exact evolutionary history.

#### 5. Mapping the phylogenetic tree onto the PCoA result

The core algorithm of PhyloMap is to map the phylogenetic tree onto the two-dimensional coordinates calculated by PCoA. We adopted a multidimensional scaling method (MDS) similar to "Sammon's mapping" [22], but a few changes have been made to fit our specific problem.

A phylogenetic tree has two types of nodes:

• Leaf nodes: nodes that do not have any children; each node represents a sequence.

• Inner nodes: nodes have children nodes and a parent node. The root node of the tree can be considered a special inner node that has no parent node.

Each leaf node corresponds to one point in the two-dimensional PCoA result. The positions of these points are fixed, which means the coordinates of the leaf nodes are predefined and cannot be changed when drawing the tree. If we want to preserve the edge length between nodes, only the inner nodes can be moved. Unlike other MDS problems where the distances of one data point to all other data points are known, in PhyloMap each inner node is only constrained by three other nodes: one parent node and two children nodes.

We first define an error function *E*_*s *_similar to "Sammon's mapping":(3)

where *s *is a scaling factor that compensates for the distance difference between the tree space and the PCoA space (if the same distance measurement is used both in PCoA and tree building, then *s *= 1),  is the edge length between node *s *and node *j *in the tree, and *d*_*ij *_is the distance between node *i *and node *j *in the 2D PCoA result.

**Figure 2:**
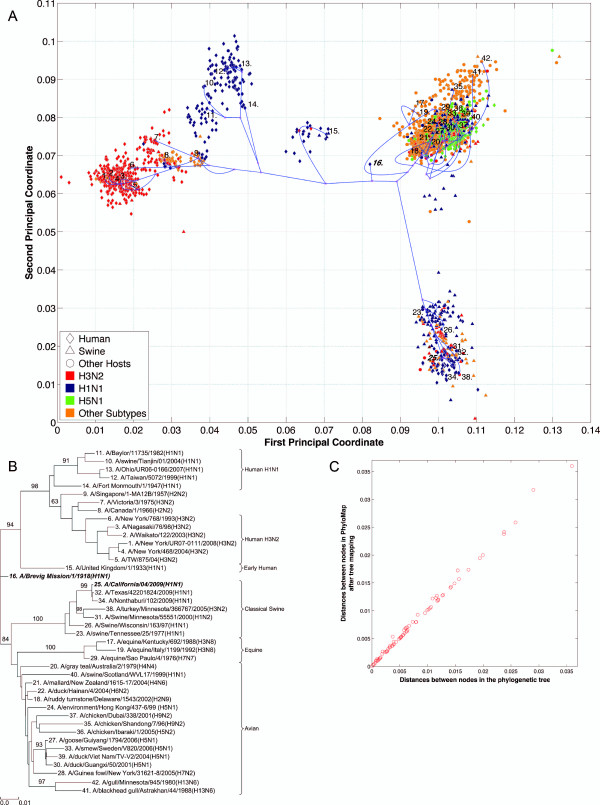
**NP PhyloMap**. (*A*) The PhyloMap for 2984 NP protein sequences. Each spot in the plot corresponds to one sequence, and the first two dimensions represent 56.7% of the total variation. The phylogenetic tree mapped onto the plot is shown in (*B*). The mapping error is 0.00259. The strain names that stand for the numbers in the plot are shown in the phylogenetic tree in (*B*). (*B*) The NJ tree of NP protein sequences built using distances inferred by the JTT model; 40 sequences have been selected by PhyloMap as data centers, the other two sequences (in bold italics) have been added manually. This tree has been mapped onto the PCoA result as shown in (*A*). Bootstrap values (1000 replications) for key nodes are shown. The tree was annotated using "TreeGraph 2" [58]. (*C*) The relationships of the distances between nodes in the original phylogenetic tree (*B*) and in the PhyloMap after mapping (*A*). Correlation coefficient: 0.998. Errors before Bezier curve compensation: 0.0496, after Bezier curve compensation: 0.00259. The errors after Bezier curve compensation are caused by the distances that are longer in the PhyloMap than in the original tree.

The algorithm will then employ gradient descent on the inner nodes to minimize* E*_*s*_. The distance *d*_*ij *_defined between node *i *and *j *is the straight-line distance. However, in our problem, the straight-line distance can only generate poor results, either large *E*_*s *_or a plot that is difficult to inspect visually. This is because the leaf nodes cannot move and, hence, all the distance constraints have to be satisfied by the inner nodes. If the inner nodes only explore a small space, which will provide attractive visual results,* E*_*s *_might be too large to accurately preserve the tree distances. To solve this problem, we use the Bezier curve [28] to compensate for the distances that are shorter than in the original tree. In this case, if the distances are shorter, they can be exactly preserved in the PhyloMap. Only the distances larger than in the original tree will contribute to the error (Figure 2C). So in the gradient descent procedures, we use a strategy which tries to keep most of the straight-line distances shorter than in the original by updating the longer distances more frequently than the shorter ones. The error function *E*_*b *_after Bezier curve compensation is defined as:(4)

where  is the length of the Bezier curve between node *i *and node *j*.

The algorithm can be summarized as follows:

**Input**: tree: *T*; leaf-node coordinates: *C*_*leaf*_; scaling factor: *s*; max. number of iterations: *maxiters*; error *e*.

**Output**: all node coordinates *C*_*node*_, corresponding Bezier curve control point *C*_*bezier *_and error *E*_*b *_after Bezier curve compensation.

1: *Du *:= calculate the desired distance matrix using all nodes in *T*.

2: *C*_*node *_:= randomly initializing the coordinates of the inner nodes and attach *C*_*leaf*_.

3: *D*_*s *_:= calculate the actual distance matrix using *C*_*node*_.

4: **while ***maxiters *is not reached or *e*_*i *_≤ *e*

5: **for each **inner node

6: update the coordinate of the inner node using gradient decent once every five iters.

7: update the coordinates of the inner node using gradient decent only if

there exists at least one edge connected to this node with  four times every five iters.

8: update *D*_*s *_using the new coordinates.

9: **end for each**

10: *e*_*i *_:= calculate error using equation (3).

11: **end while**

12: **for each **

13: *C*_*bezier *_:= calculate the Bezier curve control point so that .

14: **end for each**

15:*E*_*b *_:= calculate error using equation (4).

### Influenza A virus genome data

We compiled a data set containing 74,309 sequences of influenza A virus internal proteins as available from the NCBI database [29] on 03-01-2010 (as summarized in Table 1). We defined strict rules [30] for data validation to ensure a high quality of our dataset. Each sequence included in the data set is complete or nearly complete.

**Table 1:** Number of protein sequences used in the data set

	PB2	PB1	PA	NP	M1	M2	NS1	NS2
No. of sequences	8397	8577	8522	8590	11258	10111	9982	8872
No. of non-redundant sequences	4384	4022	4173	2984	1496	2016	3734	1650

All eight gene products were aligned separately using MUSCLE [31], and the alignment results were curated manually to assure a high quality such that gaps were minimal. For calculating the distance matrix (described above), protein sequences were used. The reason to use protein instead of nucleotide sequences is that while at the nucleotide level, two sequences may vary greatly, they may be very close at the amino-acid level due to functional restraints [15,19]; thus, the distance between two amino-acid sequences is more relevant for the assessment of their functional differences. For most of the internal genes, around half of the protein sequences are redundant. Hence, only one of a set of identical sequences was used to compose the data set as the input of the PhyloMap.

## Results

### PhyloMap reduces the risk of misinterpretation

**Figure 3:**
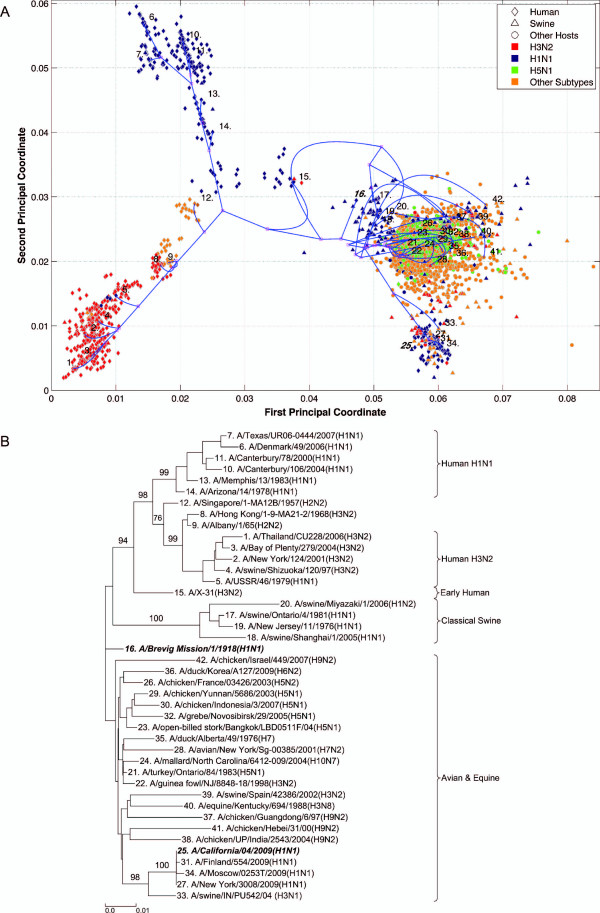
**PB2 PhyloMap**. (*A*) The PhyloMap for 4384 PB2 protein sequences. Each spot in the plot corresponds to one sequence, and the first two dimensions represent 49.6% of the total variation. The phylogenetic tree mapped onto the plot is shown in (*B*). The mapping error is 0.00527. The strain names that stand for the numbers in the plot are shown in the phylogenetic tree in (*B*). (*B*) The NJ tree of PA protein sequences built using distances inferred by the JTT model, 40 sequences have been selected by PhyloMap as data centers, the other 2 sequences (in bold italics) have been added manually. This tree has been mapped onto the PCoA result as shown in (*A*). Bootstrap values (1000 replications) for key nodes are shown.

**Figure 4:**
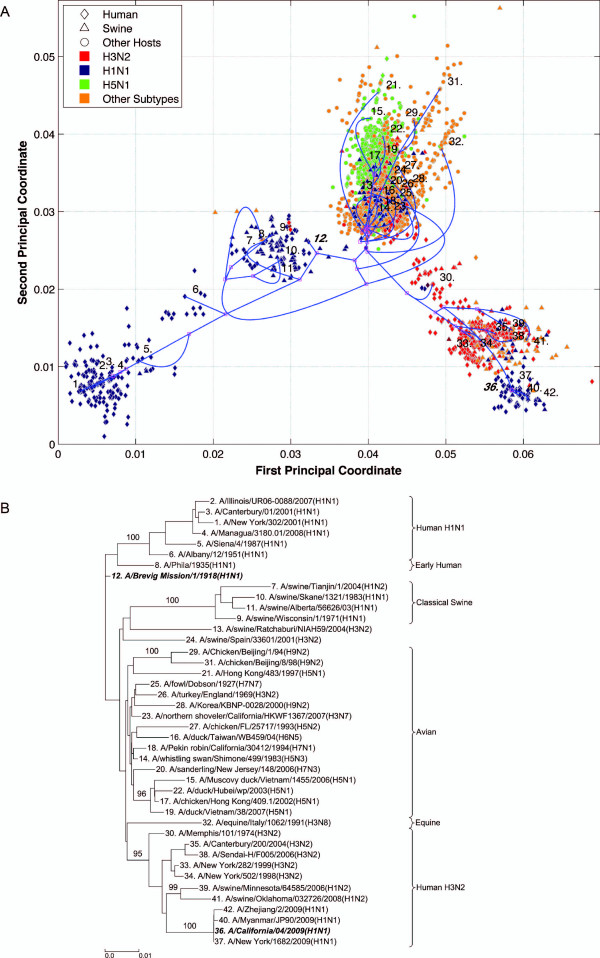
**PB1 PhyloMap**. (*A*) The PhyloMap for 4022 PB1 protein sequences. Each spot in the plot corresponds to one sequence, and the first two dimensions represent 41.4% of the total variation. The phylogenetic tree mapped onto the plot is shown in (*B*). The mapping error is 0.00261. The strain names that stand for the numbers in the plot are shown in the phylogenetic tree in (*B*). (*B*) The NJ tree of PB1 protein sequences built using distances inferred by the JTT model, 40 sequences have been selected by PhyloMap as data centers, the other 2 sequences (in bold italics) have been added manually. This tree has been mapped onto the PCoA result as shown in (*A*). Bootstrap values (1000 replications) for key nodes are shown.

**Figure 5:**
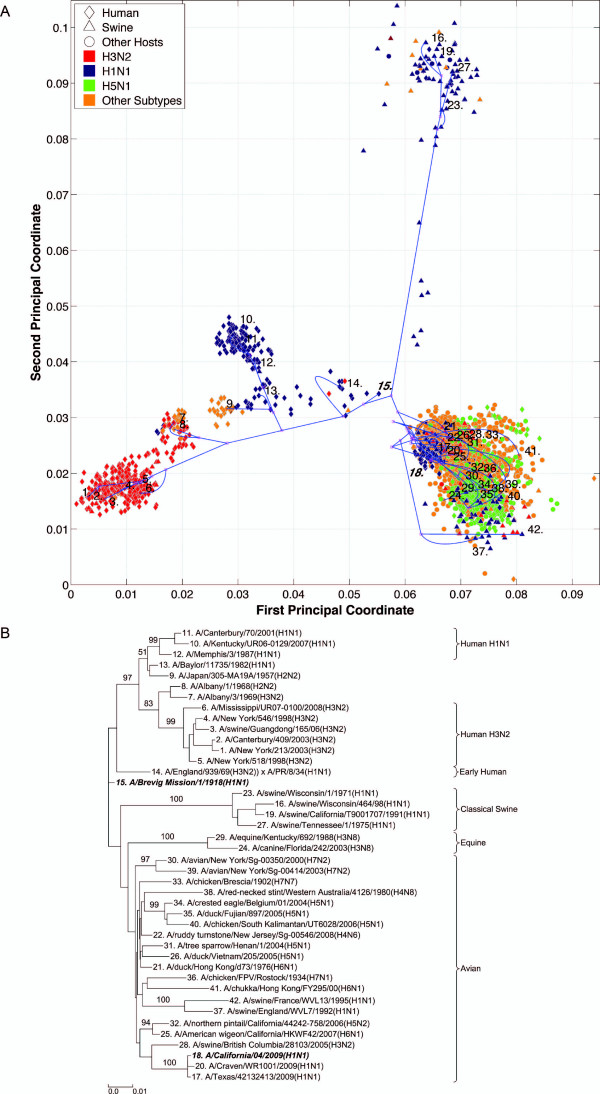
**PA PhyloMap**. (*A*) The PhyloMap for 4173 PA protein sequences. Each spot in the plot corresponds to one sequence, and the first two dimensions represent 47.6% of the total variation. The phylogenetic tree mapped onto the plot is shown in (*B*). The mapping error is 0.00253. The strain names that stand for the numbers in the plot are shown in the phylogenetic tree in (*B*). (*B*) The NJ tree of PA protein sequences built using distances inferred by the JTT model, 40 sequences have been selected by PhyloMap as data centers, the other 2 sequences (in bold italics) have been added manually. This tree has been mapped onto the PCoA result as shown in (*A*). Bootstrap values (1000 replications) for key nodes are shown.

**Figure 6:**
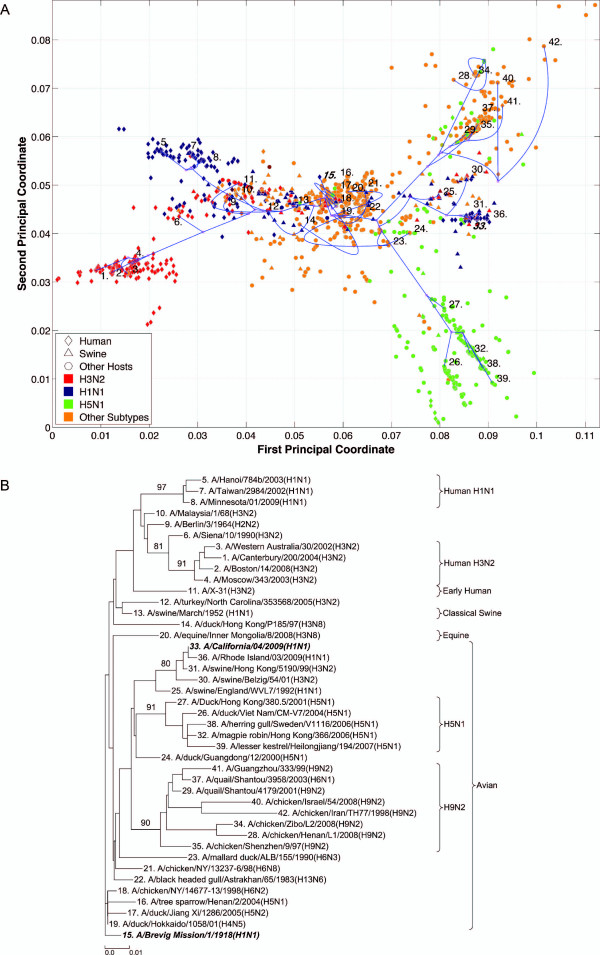
**M1 PhyloMap**. (*A*) The PhyloMap for 1496 M1 protein sequences. Each spot in the plot corresponds to one sequence, and the first two dimensions represent 43.3% of the total variation. The phylogenetic tree mapped onto the plot is shown in (*B*). The mapping error is 0.00531. The strain names that stand for the numbers in the plot are shown in the phylogenetic tree in (*B*). (*B*) The NJ tree of M1 protein sequences built using distances inferred by the JTT model, 40 sequences have been selected by PhyloMap as data centers, the other 2 sequences (in bold italics) have been added manually. This tree has been mapped onto the PCoA result as shown in (*A*). Bootstrap values (1000 replications) for key nodes are shown.

**Figure 7:**
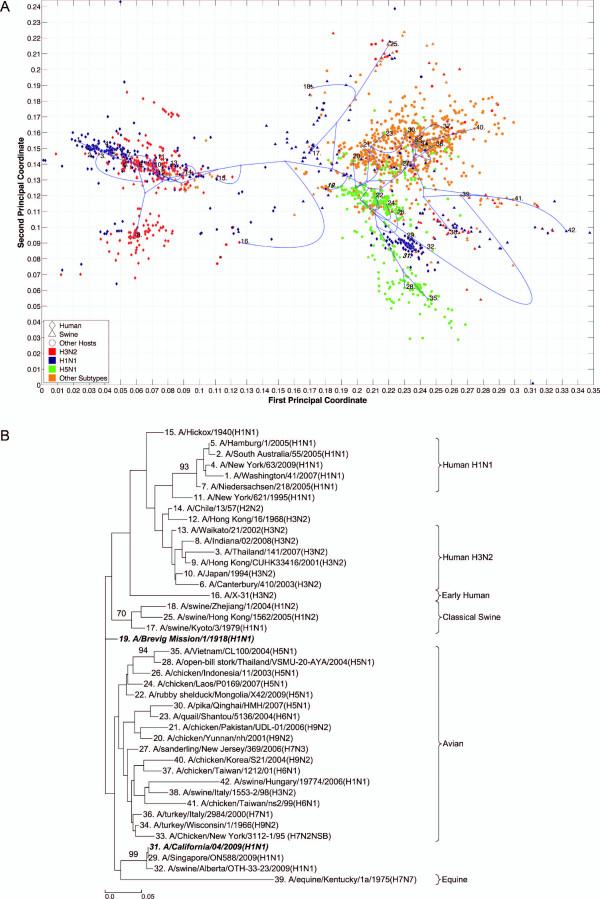
**M2 PhyloMap**. (*A*) The PhyloMap for 2016 M2 protein sequences. Each spot in the plot corresponds to one sequence, and the first two dimensions represent 44.9% of the total variation. The phylogenetic tree mapped onto the plot is shown in (*B*). The mapping error is 0.013. The strain names that stand for the numbers in the plot are shown in the phylogenetic tree in (*B*). (*B*) The NJ tree of M2 protein sequences built using distances inferred by the JTT model, 40 sequences have been selected by PhyloMap as data centers, the other 2 sequences (in bold italics) have been added manually. This tree has been mapped onto the PCoA result as shown in (*A*). Bootstrap values (1000 replications) for key nodes are shown.

**Figure 8:**
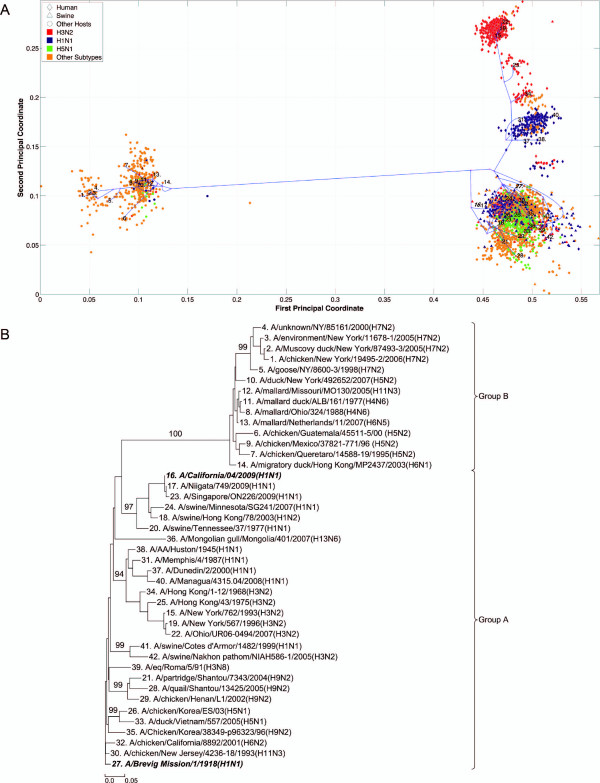
**NS1 PhyloMap**. (*A*) The PhyloMap for 3734 NS1 protein sequences. Each spot in the plot corresponds to one sequence, and the first two dimensions represent 60.1% of the total variation. The phylogenetic tree mapped onto the plot is shown in (*B*). The mapping error is 0.0023. The strain names that stand for the numbers in the plot are shown in the phylogenetic tree in (*B*). (*B*) The NJ tree of NS1 protein sequences built using distances inferred by the JTT model, 40 sequences have been selected by PhyloMap as data centers, the other 2 sequences (in bold italics) have been added manually. This tree is mapped onto the PCoA result as shown in (*A*). Bootstrap values (1000 replications) for key nodes are shown.

**Figure 9:**
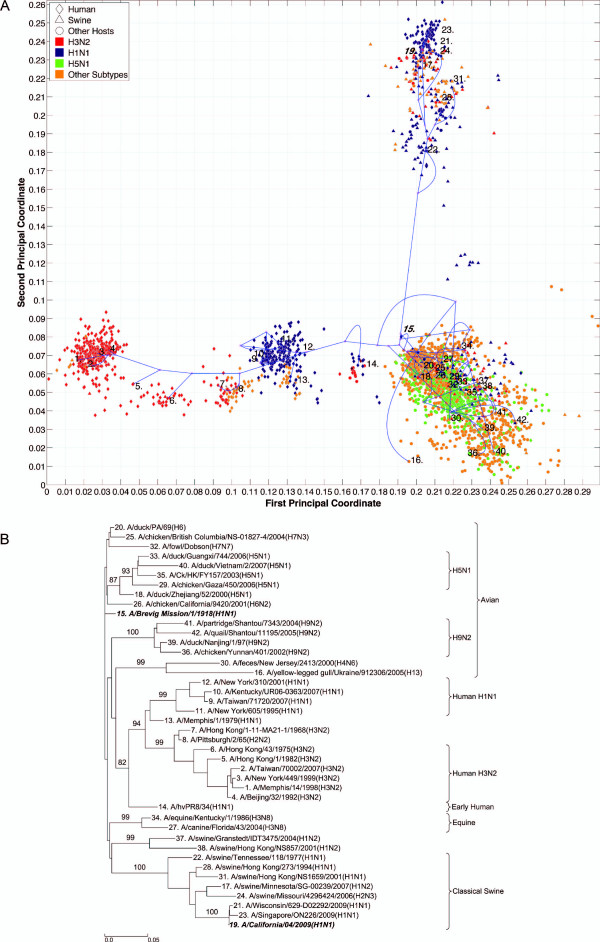
**NS1 PhyloMap excluding Group B**. (*A*) The PhyloMap for 3283 NS1 protein sequences excluding Group B. Each spot in the plot corresponds to one sequence, and the first two dimensions represent 48.2% of the total variation. The phylogenetic tree mapped onto the plot is shown in (*B*). The mapping error is 0.00252. The strain names that stand for the numbers in the plot are shown in the phylogenetic tree in (*B*). (*B*) The NJ tree of NS1 protein sequences excluding Group B built using distances inferred by the JTT model, 40 sequences have been selected by PhyloMap as data centers, the other 2 sequences (in bold italics) have been added manually. This tree has been mapped onto the PCoA result as shown in (*A*). Bootstrap values (1000 replications) for key nodes are shown.

**Figure 10:**
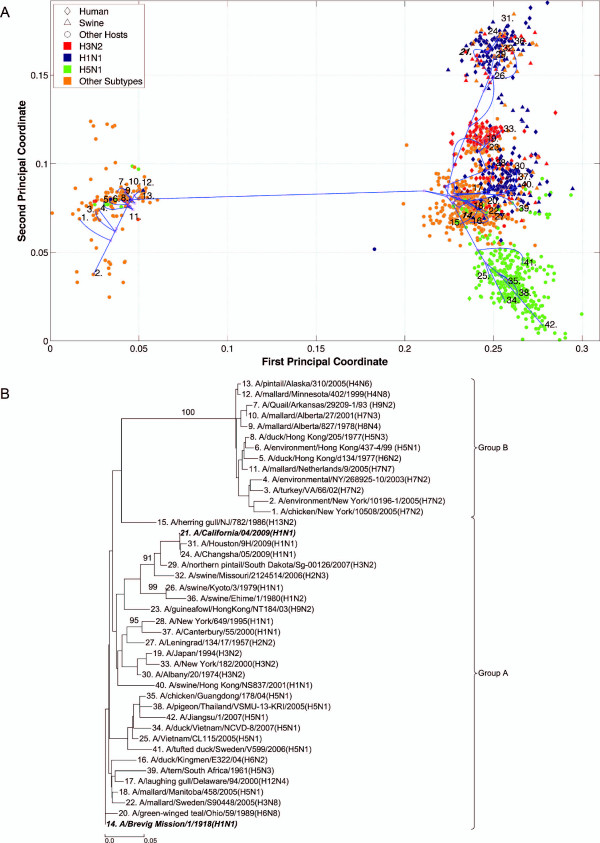
**NS2 PhyloMap**. (*A*) The PhyloMap for 1650 NS2 protein sequences. Each spot in the plot corresponds to one sequence, and the first two dimensions represent 52.7% of the total variation. The phylogenetic tree mapped onto the plot is shown in (*B*). The mapping error is 0.00485. The strain names that stand for the numbers in the plot are shown in the phylogenetic tree in (*B*). (*B*) The NJ tree of NS2 protein sequences built using distances inferred by the JTT model, 40 sequences have been selected by PhyloMap as data centers, the other 2 sequences (in bold italics) have been added manually. This tree has been mapped onto the PCoA result as shown in (*A*). Bootstrap values (1000 replications) for key nodes are shown.

**Figure 11:**
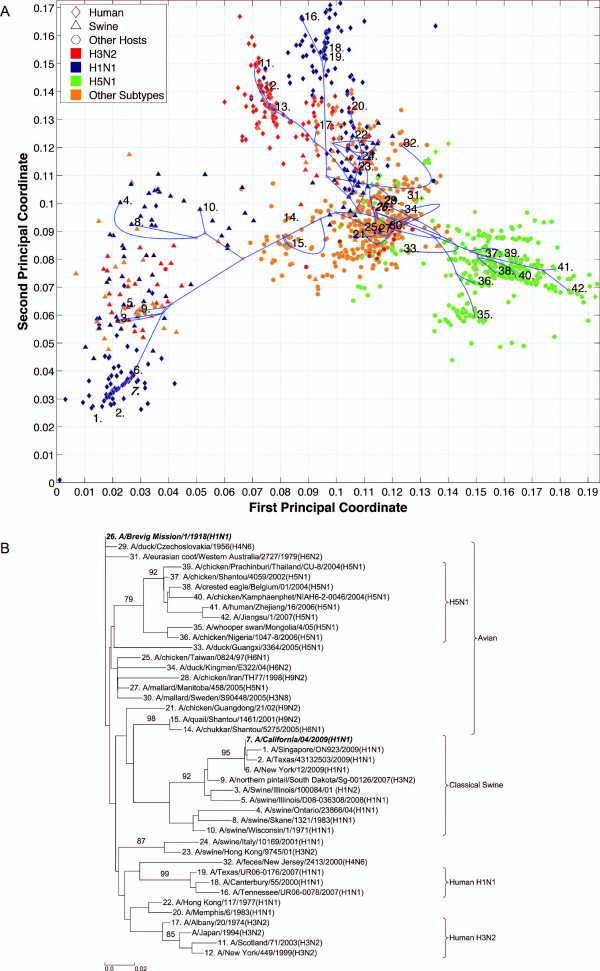
**NS2 PhyloMap excluding Group B**. (*A*) The PhyloMap for 1471 NS2 protein sequences excluding Group B. Each spot in the plot corresponds to one sequence, and the first two dimensions represent 38.7% of the total variation. The phylogenetic tree mapped onto the plot is shown in (*B*). The mapping error is 0.00427. The strain names that stand for the numbers in the plot are shown in the phylogenetic tree in (*B*). (*B*) The NJ tree of NS2 protein sequences excluding Group B built using distances inferred by the JTT model, 40 sequences have been selected by PhyloMap as data centers, the other 2 sequences (in bold italics) have been added manually. This tree has been mapped onto the PCoA result as shown in (*A*). Bootstrap values (1000 replications) for key nodes are shown.

We have generated the PhyloMap for all influenza A virus internal genes using their protein sequences, i.e. PB2, PB1, PA, NP, M1, M2, NS1, and NS2 (Figures 2, 3, 4, 5, 6, 7, 8, 9, 10 and 11). Figure 2A illustrates the results for the example of the influenza A virus NP gene. The following major lineages can be easily identified: *(i)*, seasonal human H1N1 (as shown by the data points close to "12: A/Taiwan/5072/1999(H1N1)"), *(ii)*, seasonal human H3N2 (as shown by the data points close to "2: A/Waikato/122/2003(H3N2)"), *(iii)*, early human (as shown by the data points close to "15: A/United Kingdom/1/1933(H1N1)"), *(iv)*, classical swine [32] (as shown by the data points close to "26: A/Swine/Wisconsin/163/97(H1N1)", which includes S-OIV), *(v)*, equine (as shown by the data points close to "15: A/United Kingdom/1/1933(H1N1)"), and *(vi)*, avian (as shown by the data points close to "20: A/gray teal/Australia/2/1979(H4N4)"). PhyloMap has successfully captured all major lineages of the influenza A virus NP gene that were shown to exist in a previous study [3] using sequences sampled manually.

It is obvious that PCoA alone can already identify most of the major lineages; however, without the support of the mapping tree, it fails to portray the distances between some strains. The straight-line distance between "29: A/equine/Sao Paulo/4/1976(H7N7)" and "33: A/smew/Sweden/V820/2006(H5N1)" is short, but if we follow the tree, the distance is substantially longer. The real distance may need another dimension in the PCoA to be displayed. The tree here has served to add more dimensions to the 2D PCoA plot.

While the topology of the tree is defined, different tree-drawing algorithms can generate very different tree representations. The subtrees can be arbitrarily placed by the tree-drawing algorithms [33] and can be moved up and down with a certain degree of freedom. The relationships between taxas usually cannot be clearly observed without further manually adjusting the tree. PCoA here has defined the positions of the leaf nodes in PhyloMap, which intuitively provide clustering information and the scale of their divergences. In a phylogenetic tree, some intermediate sequences would be arbitrarily placed into one of the major lineages [9]; however, with the guiding of PCoA, the intermediate position of such sequences becomes apparent. For example in Figure 2B, we might interpret the phylogenetic tree by putting the protein sequence of "9: A/Singapore/1-MA12B/1957(H2N2)" into the human H3N2 lineage if only the tree is present, but its obvious intermediate position can be clearly seen in the PhyloMap (Figure 2A). The low bootstrap value of that subtree also suggests caution should be applied when drawing conclusions from the phylogenetic tree.

### The diversity of influenza A virus internal genes

Six distinct major lineages can be identified from the PhyloMap for all genes, i.e. seasonal human H3N2, seasonal human H1N1, early human, classical swine, equine, and avian viruses. The latter have been further separated into two sublineages (western hemisphere avian lineage and eastern hemisphere avian lineage) in a previous study [3] that used nucleotide sequences, but this cannot be unambiguously observed from the PhyloMap built with protein sequences. For PB2, the triple reassortment swine strains [34,35], which include the S-OIV, form a visually separable lineage in PhyloMap (Figure 3).

The PhyloMap shows similar patterns for PB2, PA, NP, M1, and M2 (Figures 3, 5, 2, 6 and 7). The NS1 and NS2 genes are different from other genes by having a unique lineage called Group B. We can see from the PhyloMap plot (Figures 8 and 10) that NS1 and NS2 Group B has a clear boundary and is far away from other sequences, which are collectively called Group A [36]. Because of Group B, the NS1 and NS2 PhyloMap looks very different from other genes. However, if we remove Group B sequences from the NS1 and NS2 data set and recalculate the plot (Figures 9 and 11), we can see a topology similar to other genes (Figures 2, 3, 4, 5, 6 and 7). NS1 and NS2 Group B is composed of a variety of subtypes that are mostly avian strains, with only a few human and swine cases. The sample time spans the years from 1949 to 2008. However, other internal genes in the strains that contain Group B NS1 and NS2 genes do not form a separate lineage and most of them fall into the lineage of avian viruses.

PB1 also shows a pattern very different from other genes. PB1 of human H3N2 was derived from avian strains in 1968 through reassortment [3,37]. We can see from the PhyloMap that the human H3N2 virus PB1 sequences are closer to avian strains than other H3N2 genes. Moreover, PB1 shows a more conservative evolution pattern, as the genetic distances between different lineages are much smaller than for other internal genes. Another recent study also suggested the conservation of PB1 [19]. This is easy to explain, as PB1 is the catalytic subunit of the viral RNA-dependent RNA polymerase and should have a stable function in any host. A single amino-acid exchange in the functional site may abolish protein function and interrupt the viral life cycle.

The swine influenza viruses spread throughout the entire PhyloMap, further supporting the idea of swine being a "mixing-vessel" [38,39]. We also observed from the current sequenced samples that there are no avian strains containing internal gene segments from seasonal human strains. In contrast, there are many human strains carrying some internal gene segments from avian viruses. This observation combined with the seasonal human strain internal gene segments can be clearly separated from avian strains (except for PB1), suggesting that once the internal gene segments were fully adapted to man, they lost the ability to infect avian hosts.

By observing the first few dimensions of PCoA results, one can tell what are the major forces causing the data to variate from each other. We can see that the first dimension in our PCoA results on the internal genes generally reflects the host differences, and the second dimension reflects some of the subtype differences. The third dimension (not shown in the figures) further separates the swine and equine strains from others. The above observations show that the diversities of influenza A virus internal genes are mainly shaped by host differences and virus subtypes. However, using only subtype and host information is still not enough to distinguish major lineages among internal genes. For instance, the human H1N1 strains contain three major lineages: human seasonal H1N1, early human H1N1, and 2009 pandemic H1N1. These are highlighted in additional files (Additional files 1, Figure S1, Additional files 2, Figure S2, Additional files 3, Figure S3, Additional files 4, Figure S4, Additional files 5, Figure S5, Additional files 6, Figure S6, Additional files 7, Figure S7, Additional files 8, Figure S8 and Additional files 9).

### PhyloMap helps locating the origin of emerging influenza A virus

As the main patterns of influenza A internal genes can be clearly seen from the PhyloMap result, one can start to investigate the more subtle relationships of the data by zooming in onto certain clusters or adding sequences of interest into the sampling tree. The sequences of the sampling tree found by the Neural-Gas approach minimize the quadratic errors. As a result, they can well represent the diversity of the data set. When it comes to finding the origin of a new strain, the samplings can provide a good reference data set that would not miss important lineages. We have mapped the genes of 1918 "Spanish flu" ("***A/Brevig Mission/1/1918(H1N1)***") and S-OIV ("***A/California/04/2009(H1N1)***") into the PhyloMap in addition to the sampling sequences. In our sampling trees, the "Spanish flu" (internal genes) forms a separate branch and cannot be put into any major lineages. This orphan position of "Spanish flu" seems to support the previous notion that these gene segments may have been acquired from a reservoir of influenza virus that has not yet been sampled [17,18]. One can also easily identify the origin of every internal gene of S-OIV from PhyloMap: PB2, PA, M1, and M2 from avian strains; PB1 from human H3N2; NP, NS1, and NS2 from classical swine.

## Discussion

While phylogenetic tree inference methods are relatively well developed, their interpretation relies heavily on visual inspection [40]. The difficulties of analyzing a huge tree have been mainly tackled by developing sophisticated tree visualization software. Visual data exploration usually follows a three-step process [41]: overview, zoom and filter, and details-on-demand. Despite advances in the visualization software [42,43], it is very difficult to comprehend the entire tree during the overview stage. When the data set reaches a few thousand sequences, this way of phylogeny analysis becomes almost impossible. PhyloMap was developed specifically for the overview process by summarizing the main phylogeny information. Both PCoA and "Neural-Gas" can be considered data compression techniques suitable to preserve the most important information in the data. Once the main trends in the data set are identified, one can zoom in onto areas of interest, thus reducing the data set to a size that can be well visualized by traditional phylogenetic trees.

Other means of adding more information to ordination such as superimposing a minimal spanning tree and a relative neighborhood graph have been proposed by Guiller [44]. However, all those methods require using all the data points, thereby only generating unrecognizable results when the data set is large. Our proposed method can also serve as a general way of adding another layer of information to any ordination analysis of data relationships that can alternatively be described by using a tree structure.

The PCoA used here is a linear dimensionality-reduction technique [45,46]. Despite the recent advances in nonlinear dimensionality reduction, we find PCoA very suitable for PhyloMap. First, PCoA finds the greatest variance in the data set; in other words, it preserves the global pattern and this is one of the main purposes of PhyloMap. Other methods such as Isomap [45] using geodesic distance might not make too much sense in phylogenetic analysis. Methods such as LLE [46] are designed to preserve local properties which is obviously not suitable for PhyloMap. Second, PCoA is robust in the sense that it does not depend on the initiation and does not require other parameters. The well-established algorithm for solving PCoA is both computationally efficient and numerically stable. Although the phylogenetic distances inferred using some evolutionary models are not Euclidean, resulting in negative eigenvalues, in practice, those values are usually very small compared to the first few eigenvalues. Thus, they have only minor influence on the results and will not distort the main trends in the data.

In PhyloMap, we use distance-based methods to build the sampling tree. As the distances are measured in the same way both in PCoA and in the phylogenetic tree, when mapping the tree onto the PCoA result, the error can be minimized. However, the sampling tree can also be built with parsimony-based or maximum-likelihood based methods. But in such cases, the edge lengths in the tree and the 2D PCoA result might not be on the same scale. We need to estimate the scaling factor *s *in equation (4). It is very difficult to exactly estimate *s *before the mapping is made, so *s *can only be searched within a certain range (The ratio of the distance between the furthest cluster centers in the PCoA result and the corresponding length in the tree can be a good starting value). This problem does not exist in classical MDS, since all the data points during the mapping can move freely, but in PhyloMap, the leaf nodes are fixed.

The accuracy of an inferred phylogenetic tree depends on many factors such as the number of sequences, number of characters (number of aligned positions), and substitution rate. In general, the accuracy of the inferred phylogenetic tree increases while more characters are used [47,48]. However, there are also many debates on whether to increase the number of sequences or the number of characters to improve the resolution of the phylogenetic analysis. In the case that the number of available characters to build the phylogenetic tree is fixed such as for the internal genes of influenza A virus, one might choose a small number of sequences to derive the most reliable tree. There are two interesting questions connected with this approach: how to choose the sequences, i.e. which sampling methods to apply, and how many sequences are needed given the number of characters. As for the sampling, we believe that clustering methods such as Neural-Gas should be used in order to avoid bias to arise from manual sampling, although some criteria should be developed to further test the influence of different clustering methods on the accuracy of the inferred tree. But an objective way of finding the optimal number of sequences is still lacking, and further theoretical and empirical studies are needed.

## Conclusions

PhyloMap is a robust algorithm for analyzing phylogenetic relationships in large sequence data sets. It can utilize the entire data set and avoids the bias introduced by manual samplings. PhyloMap introduces two data compression techniques (dimensionality reduction and vector quantization) into phylogenetic studies to reduce the data without losing important information. The visualizations generated summarize the main phylogeny information and overcome the shortcomings of phylogenetic tree construction and ordination analysis when used alone.

There have been only a few studies targeting the phylogenetic diversity of the internal genes of influenza A virus [3,8,54]. However, the phylogenetic trees built in some of these studies only sampled a small portion of the data and therefore might not reflect the actual size and composition of the lineages, and the representative sequences might be biased [3]. PhyloMap gives a more comprehensive overall picture of the evolution of influenza A viruses and may further help define a new nomenclature system for influenza A viruses.

Research on influenza A viruses has suggested that they are constantly undergoing frequent reassortment [55,56]. However, as the overall phylogenetic relationships of the internal genes have been largely unknown so far, few studies have addressed the scale of reassortment and the patterns of segment compatibility in cases where the reassortment occurred between distant lineages [57]. Furthermore, a robust way of identifying reassorted strains is lacking. When a new strain emerges, it is a tedious job for researchers to compare different topologies of various phylogenetic trees to find the reassortment patterns. We are confident that PhyloMap can help develop new insights into the relationships between the internal genes, in order to find new means of studying reassortment.

PhyloMap is implemented in JAVA, and the source code is freely available for download at http://www.biochem.uni-luebeck.de/public/software/phylomap.html To visualize the results, some Matlab routines are also available from the above link.

## Competing interests

The authors declare that they have no competing interests.

## Authors' contributions

JZ designed and implemented the PhyloMap algorithm, analyzed the data and drafted the manuscript, AMM participated in designing the PhyloMap algorithm, TM evaluated the algorithm and drafted the manuscript, SC participated in cleaning and analyzing the data, JW participated in drafting the manuscript, RH designed the research, analyzed the data and drafted the manuscript. All authors read and approved the final manuscript.

## Supplementary Material

Additional file 1 **NP PhyloMap highlights human H1N1 influenza A virus**. The figure of NP PhyloMap highlights human H1N1 influenza A virus Click here for file

Additional file 2 **PB2 PhyloMap highlights human H1N1 influenza A virus**. The figure of PB2 PhyloMap highlights human H1N1 influenza A virus Click here for file

Additional file 3 **PB1 PhyloMap highlights human H1N1 influenza A virus**. The figure of PB1 PhyloMap highlights human H1N1 influenza A virus Click here for file

Additional file 4 **PA PhyloMap highlights human H1N1 influenza A virus**. The figure of PA PhyloMap highlights human H1N1 influenza A virus Click here for file

Additional file 5 **M1 PhyloMap highlights human H1N1 influenza A virus**. The figure of M1 PhyloMap highlights human H1N1 influenza A virus Click here for file

Additional file 6 **M2 PhyloMap highlights human H1N1 influenza A virus**. The figure of M2 PhyloMap highlights human H1N1 influenza A virus Click here for file

Additional file 7 **NS1 PhyloMap highlights human H1N1 influenza A virus**. The figure of NS1 PhyloMap highlights human H1N1 influenza A virus Click here for file

Additional file 8 **NS2 PhyloMap highlights human H1N1 influenza A virus**. The figure of NS2 PhyloMap highlights human H1N1 influenza A virus Click here for file

Additional file 9 **Figure legend**. The figure legend for Additional file 1, 2, 3, 4, 5, 6, 7 and 8. Click here for file
