# Supplementary figures and images for: PhyloMap: an algorithm for visualizing relationships of large sequence data sets and its application to the influenza A virus genome
Source: BMC Bioinformatics. 2011 Jun 20;12:248. doi: 10.1186/1471-2105-12-248 (PMC3142226; doi:10.1186/1471-2105-12-248)

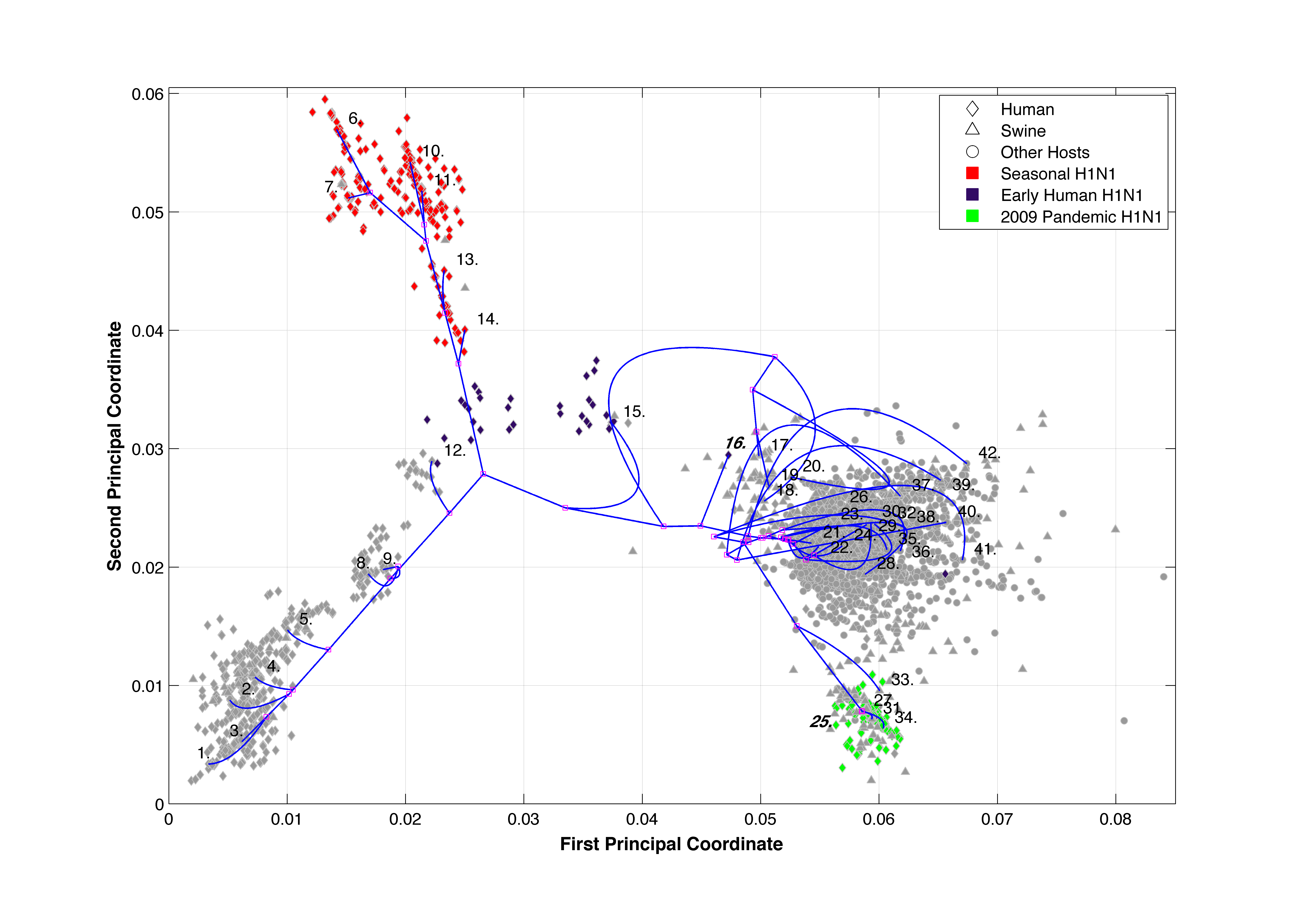

Supplement: Additional file 2 — PB2 PhyloMap highlights human H1N1 influenza A virus. The figure of PB2 PhyloMap highlights human H1N1 influenza A virus [file 1471-2105-12-248-S2.TIFF]

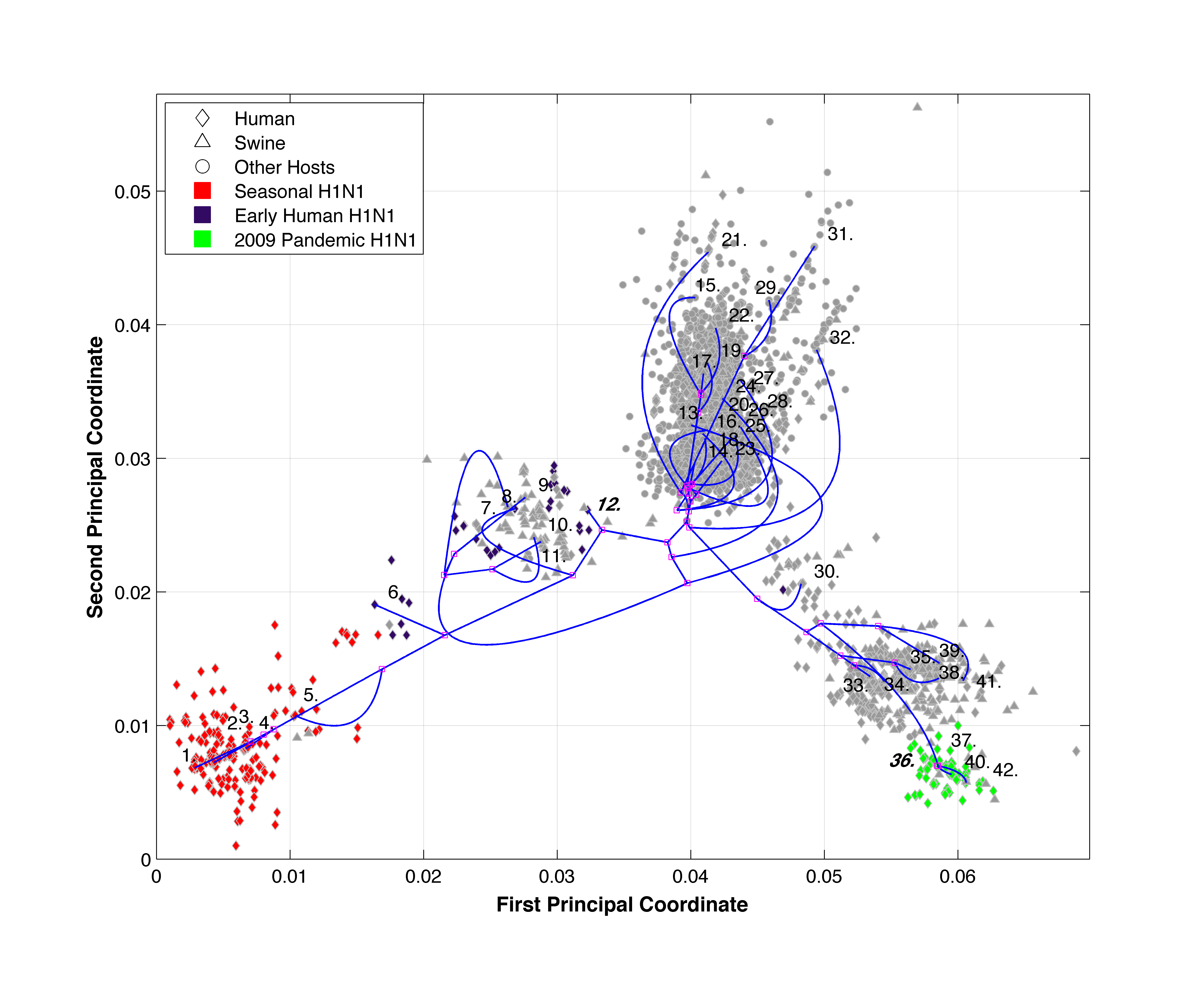

Supplement: Additional file 3 — PB1 PhyloMap highlights human H1N1 influenza A virus. The figure of PB1 PhyloMap highlights human H1N1 influenza A virus [file 1471-2105-12-248-S3.TIFF]

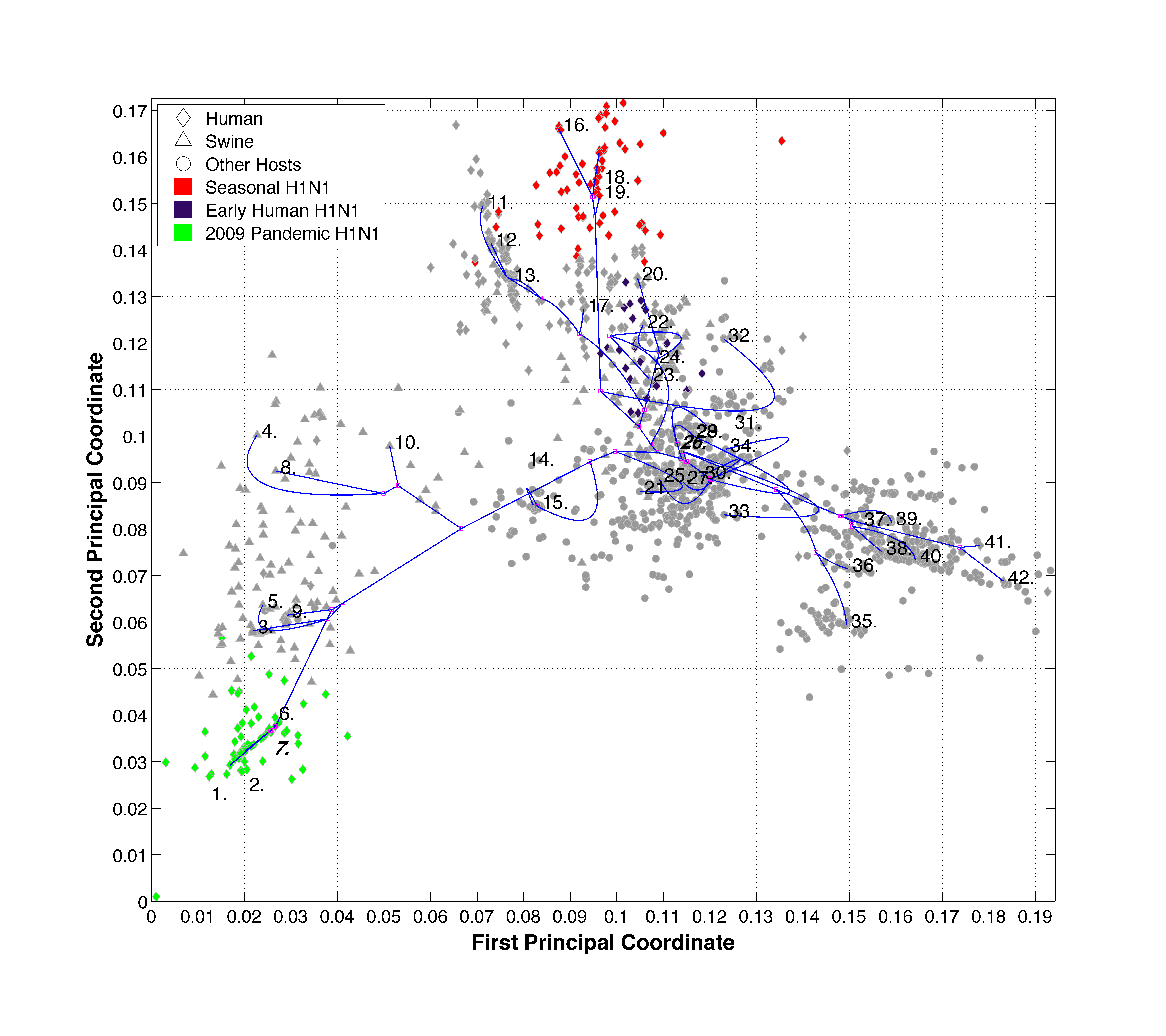

Supplement: Additional file 8 — NS2 PhyloMap highlights human H1N1 influenza A virus. The figure of NS2 PhyloMap highlights human H1N1 influenza A virus [file 1471-2105-12-248-S8.TIFF]
