# Supplementary material for: PhyloMap: an algorithm for visualizing relationships of large sequence data sets and its application to the influenza A virus genome
Source: BMC Bioinformatics. 2011 Jun 20;12:248. doi: 10.1186/1471-2105-12-248 (PMC3142226; doi:10.1186/1471-2105-12-248)
Supplement: Additional file 9 — Figure legend. The figure legend for Additional file 1, 2, 3, 4, 5, 6, 7 and 8. [file 1471-2105-12-248-S9.DOC]

Fig. S1

NP PhyloMap highlights human H1N1 influenza A virus. The strain names that stand for the numbers in the plot are the same in Fig. 2.

Fig. S2

PB2 PhyloMap highlights human H1N1 influenza A virus. The strain names that stand for the numbers in the plot are the same in Fig. 3.

Fig. S3

PB1 PhyloMap highlights human H1N1 influenza A virus. The strain names that stand for the numbers in the plot are the same in Fig. 4.

Fig. S4

PA PhyloMap highlights human H1N1 influenza A virus. The strain names that stand for the numbers in the plot are the same in Fig. 5.

Fig. S5

M1 PhyloMap highlights human H1N1 influenza A virus. The strain names that stand for the numbers in the plot are the same in Fig. 6.

Fig. S6

M2 PhyloMap highlights human H1N1 influenza A virus. The strain names that stand for the numbers in the plot are the same in Fig. 7.

Fig. S7

NS1 PhyloMap excluding Group B highlights human H1N1 influenza A virus. The strain names that stand for the numbers in the plot are the same in Fig. 9.

Fig. S8

NS2 PhyloMap excluding Group B highlights human H1N1 influenza A virus. The strain names that stand for the numbers in the plot are the same in Fig. 11.
